# Deletion of C7L and K1L Genes Leads to Significantly Decreased Virulence of Recombinant Vaccinia Virus TianTan

**DOI:** 10.1371/journal.pone.0068115

**Published:** 2013-07-01

**Authors:** Zheng Liu, Shuhui Wang, Qicheng Zhang, Meijuan Tian, Jue Hou, Rongmin Wang, Chang Liu, Xu Ji, Ying Liu, Yiming Shao

**Affiliations:** 1 Division of Research on Virology and Immunology, State Key Laboratory for Infectious Disease Prevention and Control, National Center for AIDS/STD Control and Prevention (NCAIDS), China CDC, Beijing, China; 2 Key Laboratory of Molecular Microbiology and Biotechnology (Ministry of Education) and Key Laboratory of Microbial Functional Genomics (Tianjin), College of Life Sciences, Nankai University, Tianjin, China; 3 Division of Infectious Diseases & HIV Medicine, Case Western Reserve University, Cleveland, Ohio, United States of America; 4 Clinical Laboratory, Zhaoyuan CDC, Zhaoyuan, China; George Mason University, United States of America

## Abstract

The vaccinia virus TianTan (VTT) has been modified as an HIV vaccine vector in China and has shown excellent performance in immunogenicity and safety. However, its adverse effects in immunosuppressed individuals warrant the search for a safer vector in the following clinic trails. In this study, we deleted the C7L and K1L genes of VTT and constructed six recombinant vaccinia strains VTT△C7L, VTT△K1L, VTT△C7LK1L, VTKgpe△C7L, VTKgpe△K1L and VTT△C7LK1L-gag. The pathogenicity and immunogenicity of these recombinants were evaluated in mouse and rabbit models. Comparing to parental VTT, VTT△C7L and VTT△K1L showed significantly decreased replication capability in CEF, Vero, BHK-21 and HeLa cell lines. In particular, replication of VTT△C7LK1L decreased more than 10-fold in all four cell lines. The virulence of all these mutants were decreased in BALB/c mouse and rabbit models; VTT△C7LK1L once again showed the greatest attenuation, having resulted in no evident damage in mice and erythema of only 0.4 cm diameter in rabbits, compared to 1.48 cm for VTT. VTKgpe△C7L, VTKgpe△K1L and VTT△C7LK1L-gag elicited as strong cellular and humoral responses against HIV genes as did VTKgpe, while humoral immune response against the vaccinia itself was reduced by 4-8-fold. These data show that deletion of C7L and K1L genes leads to significantly decreased virulence without compromising animal host immunogenicity, and may thus be key to creating a more safe and effective HIV vaccine vector.

## Introduction

Vaccinia virus (VACV), a member of the Poxvirus family, is one of the most common viral vectors employed in vaccines against various pathogens and diseases. VACV is able to carry a large range of foreign genes and has unique immunological properties in eliciting long-term protective humoral and cell-mediated immune responses [1,2]. The RV144 Phase III clinical trials (“Thailand trial”), using the recombinant poxvirus vector ALVAC-HIV (vCP1521) prime and AIDSVAX B/E boost, induced effective T cell responses and antibodies [3–6]. In China, vaccinia virus TianTan strain (VTT) was used as a vaccine for millions of Chinese during the global smallpox eradication campaign before 1980. More recently, VTT has been used as a replicating vector in the development of several novel vaccines, including rabies, HIV, and influenza, the safety of which have all been confirmed in animal models [7–9]. For the HIV vaccine, the *gag, pol*, and *env* genes of HIV-1 B’/C CN54 strain were inserted into VTT. This recombinant vector has completed Phase I clinical trials in China, and Phase II is currently in starting stage [10–13]. And a safer and more effective HIV vaccine will be needed in the following Phase iii clinic trial.

Despite its frequent usage in the past, the safety of this viral vector remains to be optimized. It has been reported that intranasal inoculation of VTT may cause significant body weight loss, and can even be lethal in mice after intracranial inoculation [14,15]. It is therefore necessary to generate a safer, more attenuated VTT-based vaccine vector. Many approaches have been applied to enhance the safety of poxvirus vector, such as to attenuate virulence related genes and limit replication in human and other mammalian cell lines. These efforts have given rise to the modified vaccinia virus Ankara (MVA), NYVAC, canarypox (ALVAC), and fowlpox (FP9). Among these, highly-attenuated modified vaccinia Ankara (MVA) lost 15% of its parental genome, including genes that regulate viral host range and evasion of host immune response, and NYVAC has undergone deletion of 18 open reading frams (ORFs). These vectors have been shown to be safe in preclinical and clinical trials, and their genetic alteration may point to a way to improve the safety of VTT [16–19].

Among the 18 ORF deletions in NYVAC, the C7L and K1L genes are worthy of particular attention. VACV has a very broad host range among the mammalian and avian cells, and can replicate in rabbit, pig, murine, monkey and human host cells. C7L and K1L are important genes regulating the virus’ host range, including the infection capability of VACV in human cells [20]. Possessing a copy of either K1L or C7L is sufficient for VACV to overcome replication restriction in pig kidney cells, and the K1L gene, but not C7L, allows VACA to replicate in rabbit kidney cells [21–23]. Deletion both of these genes leads to deficient replication on mammalian cells and decreased virulence in mice [24–27].

In our lab strains, the C12L, A53R, B8R and A33R genes of VTT have been deleted, which are genes associated with immunomodulatery or packaging functions of the virus [13,28], few data are available about the role of C7L and K1L in the safety and immunogenicity in VTT and VTKgpe. In order to obtain a better HIV vaccine, we constructed six recombinant strains with deletion of the C7L and/or K1L genes, and tested these strains for replication dynamics, virulence and immunogenicity in mouse and rabbit models.

## Materials and Methods

All animal experiments were conducted in accordance with the guidelines of Laboratory Animal Center of China CDC and National Center for AIDS/STD Control and Prevention. All procedures for animal use and care were approved by the National Center for AIDS/STD Control and Prevention Institutional Committee on Laboratory Animals.

### Cells and viruses

CEF cells (Chicken Embryo Fabric) were cultured at 37°C under 5% CO_2_ in Eagle’s medium supplemented with 10% fetal bovine serum (FBS, Sijiqing, China). Human cervical carcinoma HeLa cells, BHK-21 cells and Vero cells were cultured in Dulbecco’s Modified Eagle Medium (DMEM, Hyclone, USA) supplemented with 10% FBS.

VTT was provided by National Vaccine and Serum Institute and VTKgpe is a recombinant VTT vector expressing HIV-1B’/C CN54 strain *gag, pol* and *env* genes [7,13]. Both VTT and VTKgpe were propagated in CEF cells.

### Recombinant virus construction

The shuttle plasmids pMD18-T-C7L and pMD18-T-K1L each carries the start- and end-flanking sequences of the VTT C7L ORF or the K1L ORF, respectively. Instead of the ORFs, however, these fragments flank a green fluorescence protein (GFP) gene and P11 promoter sequence essentially deleting the C7L or K1L ORF. The recombinant viruses VTT△C7L and VTT△K1L were constructed by homologous recombination. Briefly, CEFs were infected with VTT at MOI=1, after incubated at 37°C for one hour, the viruses were removed and the cells were washed three times and then transfected with plasmid pMD18-T-C7L or pMD18-T-K1L. The green fluorescence-positive recombinant viruses were screened by plaque purification. After 3-5 rounds purification, the recombinant viruses VTT△C7L and VTT△K1L were obtained. VTT△C7LK1L was constructed by transfecting VTT△C7L-infected CEF cells with shuttle plasmid pLW73-K1L, which carries a K1L ORF knockout using a LacZ gene insertion. LacZ-positive plaques were screened through culture on medium containing X-Gal and purified as above.

Plasmid pSC65-gagpolenv contained HIV-1 B’/C CN54 strain *gag, pol* and *env* genes, and the LacZ gene is in the middle of the two fragments of TK region. VTKgpe△C7L or VTKgpe△K1L was constructed by transfecting CEF cells infected with VTT△C7L or VTT△K1L one hour before. LacZ-positive plaques were screened and purified as above. The plasmid pLY-Gag contained gag gene controlled by a pE/L promoter and a red fluorescence gene between fragments of I8R and G1L, VTT△C7LK1L-gag was obtained by transfecting CEF cells infected with VTT△C7LK1L, and red fluorescence-positive recombinant viruses were screened by plaque purification following fluorescence microscope inspection.

### PCR

The deletions of C7L and K1L genes were determined by PCR using primer pairs ﬂanking the deletion region. PCR primers used for this test were: 5’-CCGGAATTCCGGACACGATAGGTCAGAAGATAATG-3’ (sense) and 5’-CCCAAGCTTGGGATATAAATGCGATGTCATTCGACAG-3’ (antisense) for deletion of C7L; 5’-CCGGAATTCCGGCCGCTGAGTGGTAAACAACAGAAC-3’ (sense) and 5’-CCCAAGCTTCCGAAATGATGGCTCCTAGTATG-3’ (antisense) for deletion of K1L. PCR ampliﬁcation products were tested by agarose gel electrophoresis.

### Western blot analysis

The VTKgpe, VTKgpe△C7L, VTKgpe△K1L or VTT△C7LK1L-gag plasmids were each used to infect one well of a six well plate of CEF cells at MOI of 5. Cells were harvested at 24 hours, and treated with 0.5 ml RIPA buffer with 1% PMSF on ice for 10 minutes.

Run a 10% SDS-PAGE with cell lysates, transferred with IBlot dry system (Invitrogen, USA). Blocked the PVDF membrane with 5% non-fat dry milk in PBS for a hour, then added 1: 2000 dilution of mouse anti-p55 or anti-gp120 antibodies, incubated for 1 hour with gently waggle. PVDF membrane was washed with PBST and added 1: 20000 dilution of anti-mouse IgG with HRP for 1 hour with gently waggle. Washed with PBST and detected by using chemilluminescence (Pierce, USA).

### Growth curve analysis

Monolayers of CEF cells, HeLa cells, BHK-21 cells and Vero cells grown in six-well plates were infected with VTT, VTT△C7L, VTT△K1L or VTT△C7LK1L at MOI of 0.05. 2 hours following infection, cells was washed once, and media was newly added with 2% FBS. At 0 h, 6 h, 12 h, 24 h, 36 h and 48 h post infection, cells were collected with 1ml media, frozen and thawed three times, then sonicated for 3-5 seconds; the resulting supernatant was added to CEF cells, and virus yield for that timepoint was determined by plaque assay in the CEF cells.

### Virulence assay in Mice

For intracranial infection assay, groups of 3 week-old female BALB/c mice (n=16) were inoculated with 3×10^4^ pfu/20µl of VTT, VTT△C7L, VTT△K1L or VTT△C7LK1L. In each group, 10 mice were observed daily and survival was recorded until 10 days post infection. The remaining 6 mice were sacrificed at day 3 and 5 post-infection (n=3), and the brain was grinded and titred on CEF cells by plaque assay.

To characterize virulence in mature mice, groups of 6 week-old female BALB/c mice were infected intranasally with a dose of 10^5^ pfu of VTT, VTT△C7L, VTT△K1L or VTT△C7LK1L (n=6). Mice were weighted daily for 20 days.

### Virulence assay in rabbits

VTT, VTT△C7L, VTT△K1L or VTT△C7LK1L were diluted to 10^4^, 10^5^, 10^6^ or 10^7^ pfu per 100 µl. New Zealand Rabbit of ~2.5 kg were shaved on the back and inoculated intradermally with 100 µl of viruses per site. On day 4 and 7 post-infection, the diameters of erythema and ulcerations were measured by vernier caliper and recorded.

### Immunization

To evaluate the immunogenicity of vectors, groups of 6 week-old female BALB/c mice were infected intramuscularly (IM) with a dose of 10^7^ pfu of VTT, VTT△C7L, VTT△K1L or VTT△C7LK1L (n=5). 4 weeks post-infection, mice were sacrificed, and their spleen and blood serum were processed for ELISPOT assay and ELISA assay, respectively.

To evaluate the immunogenicity of HIV antigens, 6 week-old female BALB/c mice were inoculated IM with 10^7^ pfu of VTKgpe, VTKgpe△C7L, VTKgpe△K1L or VTT△C7LK1L-gag at weeks 0 and 8. One week after the final vaccination, mice were sacrificed and spleen and blood serum were assayed as above.

### IFN-γ and IL-2 ELISPOT assay

The cellular immune response in mice was determined by ELISPOT assay. IFN-γ ELISPOT plates (BD Biosciences, USA) were coated with 100 µl anti-murine IFN-γ capture antibody overnight at 4°C, and subsequently blocked for 2 hours using PRIM 1640 with 10% FBS at room temperature. 5×10^5^ splenocytes were added to each well in duplicates and incubated with complete PRIM 1640 containing 10% FBS and 5 µg/ml viral peptides (final concentration) for 20 hours at 37°C. The viral peptides were: HIV-1 peptide gag (AAMQILKDTINEEAA) or env (GKEVHNVWATHACVPTDPNP, SELYKYKVVEIKPLGIAPTA, QQSNLLRAIEAQQHLLQLTV) from HIV-1 Clade B’/C or vaccinia viruse peptide E3 (VGPSNSPTF). Plates were then washed and incubated with biotinylated antimouse IFN-γ mAb, peroxidase-labeled avidin and substrate BCIP/NBT reagent sequentially. Finally, plates were air-dried and the resulting spots were counted.

IL-2 ELISPOT plates (MabTech, Sweden) were activated by 70% ethanol and washed 5 times with sterile water, then plates were coated with 100µl diluted purified anti-mouse IL-2 antibody overnight at 4°C, blocked plate using PRIM 1640 containing 10% FBS, and splenocytes and HIV peptides gag, pol, env were added as in the IFN-γ assays above. Plates were then washed and incubated with H12A1 and substrate mixture sequentially. Finally, plates were air-dried and the resulting spots were counted.

### ELISA

HIV specific antibody against the gag and env viral proteins was detected using p55 and gp120 proteins (in-house production, NCAIDS, China CDC). Briefly, 96-well microplates were coated with 0.8µg/ml p55 or gp120 proteins and incubated overnight at 4°C, then washed three times with PBST followed by blocking at 37°C for 1 hour. Mice sera were serially diluted 2-fold down each column, and plates were incubated at 37°C for 2 hours. After plates were washed three times with PBST, wells were added with 1:20000 diluted anti-mouse conjugate-HRP antibody, incubated at 37°C for 1 hour and washed three times. Plates were coloured with substrate A and B, the reaction was stopped by 50 µl of 2 M H_2_SO_4_, and results at wavelengths 450 nm and 630 nm were recorded.

Vaccinia antibody was detected using wildtype virus VTT. Plates were coated with 1×10^6^ pfu/ml VTT overnight at 4°C, and VTT was inactivated by adding 100 µl of 2% paraformaldehyde and then washed three times with PBST. Subsequent procedures were the same as those described as above.

### Vaccinia neutralizing antibody detection

VTT-specific neutralizing antibody was measured by incubating 300 µl of diluted mouse sera (1:80, 1:160, 1:320, 1:640, 1:1280) with 300 pfu VTT in 600 µl of Eagle’s medium and 2% FBS at 37°C for 2 h. 300 µl of virus-serum mixture, virus alone or media was added to appropriate wells of confluent CEF in a 24-well plate. Samples were assayed in duplicate and plaques were counted 48 h later. Titers were defined as reciprocal serum dilution that caused a 50% reduction in viral plaques.

## Results

### Construction of C7L and K1L deletion vaccinia strains with or without HIV genes

**Figure 1:**
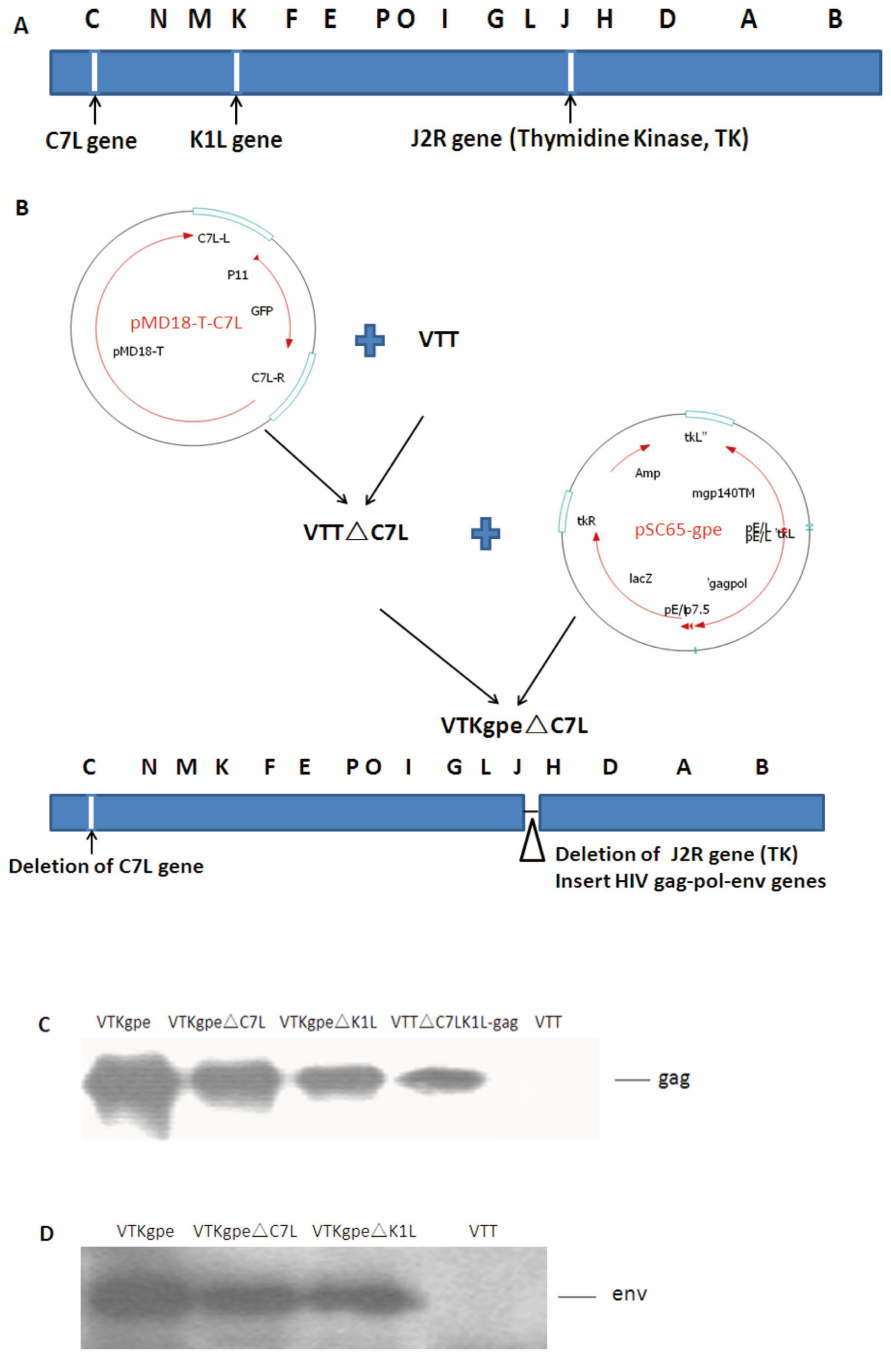
Construct of recombinant VTT and western blot analysis. (A) The deleted genes of vaccinia genome. (B) Construction of VTKgpe△C7L. (C) CEF cells were infected with indicated viruses and harvested at 24h post-infection. Lysates were prepared and analyzed by WB probed with anti-p55 antibody. (D) CEF cells were infected with indicated viruses and harvested at 24h post-infection. Lysates were prepared and analyzed by WB probed with anti-gp120 antibody.

A series of C7L and K1L mutant vaccinia have been constructed by homologous recombination techniques and named according to the deleted genes: VTT△C7L, VTT△K1L and VTT△C7LK1L. HIV-1 gag, pol, and env genes were then introduced into these mutants to generate the recombinant plasmids that serve as HIV vaccine candidates: VTKgpe△C7L, VTKgpe△K1L and VTT△C7LK1L-gag (Figure 1A 1B). Successful deletion of C7L and K1L genes were confirmed by PCR with specific primers. And adjacent genes were not affected in the recombinant vaccinia virus harboring the deletion (Sequences of adjacent genes were analyzed in Supplementary Figure S1). To verify synthesis of the corresponding proteins, we examined lysates from virus infected CEF cells by Western blot with antibodies against HIV p55 and gp120 proteins. As expected, VTKgpe△C7L, VTKgpe△K1L and VTT△C7LK1L-gag expressed 55 kD gag and 140 kD env proteins (Figure 1C 1D).

### Deletion of host range genes C7L and K1L reduce VTT’s replication capacity in CEF, BHK-21, Vero and HeLa cell lines

**Figure 2:**
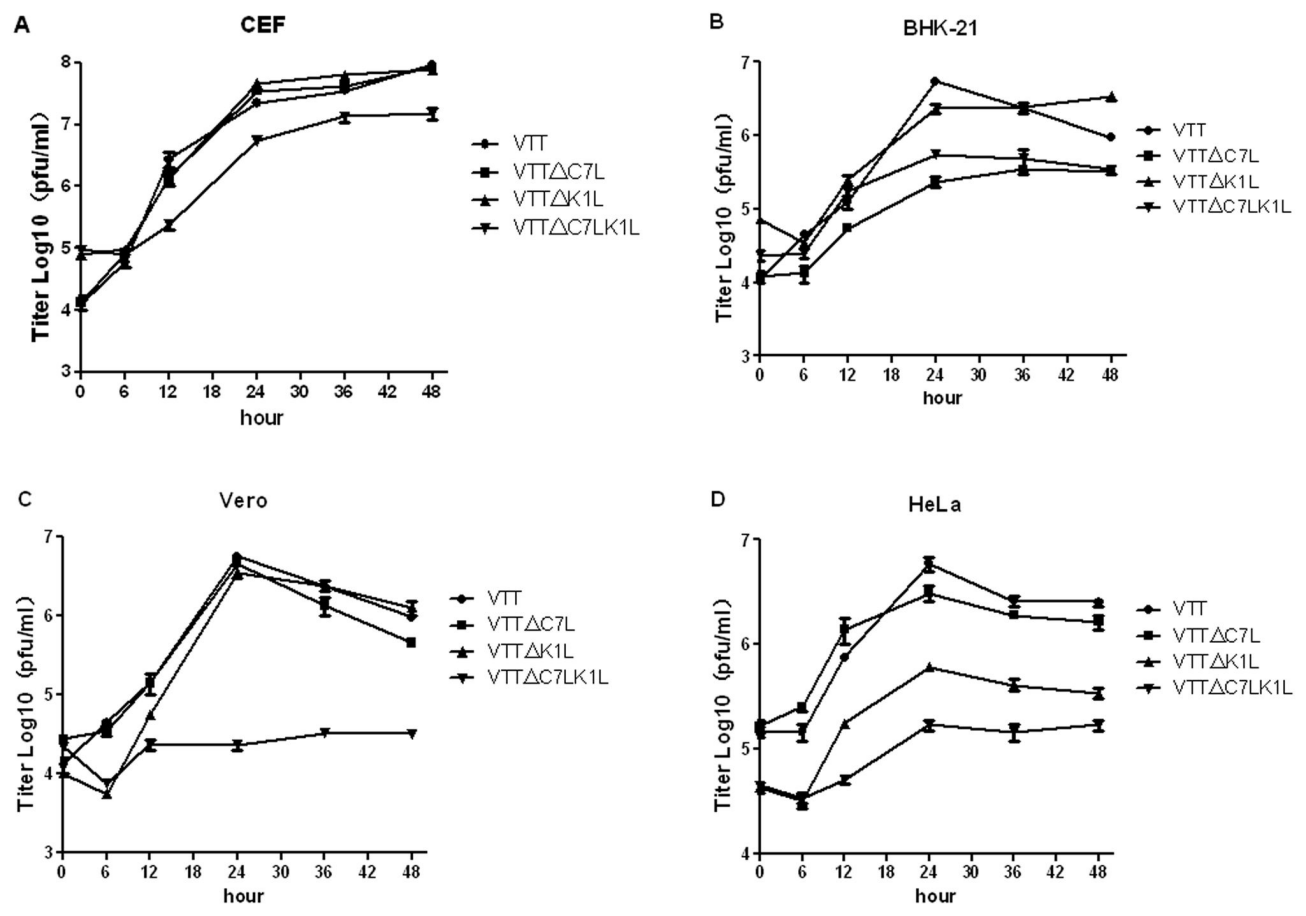
Growth curves in vitro. (A) Growth curves of the recombinant VTT in CEF cells at MOI of 0.05 pfu/cell. Virus yields at 0, 6, 12, 24, 36 and 48 h pi were determined by plaque assay on the permissive CEF cells. (B) Growth curves of the recombinant VTT in BHK-21 cells at MOI of 0.05 pfu/cell. Virus yields were determined as the former. (C) Growth curves of the recombinant VTT in Vero cells at MOI of 0.05 pfu/cell. Virus yields were determined as the former. (D) Growth curves of the recombinant VTT in HeLa cells at MOI of 0.05 pfu/cell. Virus yields were determined as the former.

To exam the influence of host range genes on the viral replication, the growth kinetics of VTT, VTT△C7L, VTT△K1L and VTT△C7LK1L were monitored in CEF, BHK-21, Vero and HeLa cell lines (Figure 2). The titer of wild type VTT reaches its peak at 48 h post-infection (p.i.) in CEFs with a 5000-fold increase from the seed concentration, and 24 hours p.i. in BHK-21, Vero and HeLa cell lines. The replication of VTT△C7L did not differ significantly from the wildtype in CEF, Vero and HeLa cells, while the replication peak dropped more than 20-fold at 24 hours p.i. in BHK-21 cells. The replication of VTT△K1L did not differ significantly in CEF and Vero cell lines, but dropped by 2.5-fold and 8-fold respectively in BHK-21 and HeLa cells. VTT△C7LK1L did not seem to replicate in Vero cells and the peak titers of VTT△C7LK1L were 9-fold, 10-fold and 33-fold lower than that of wildtype VTT in CEF, BHK-21 and HeLa cells.

### Deletion of C7L and K1L attenuates VTT’s ability to induce body weight loss and greatly attenuates VTT neurovirulence in BALB/c mouse

**Figure 3:**
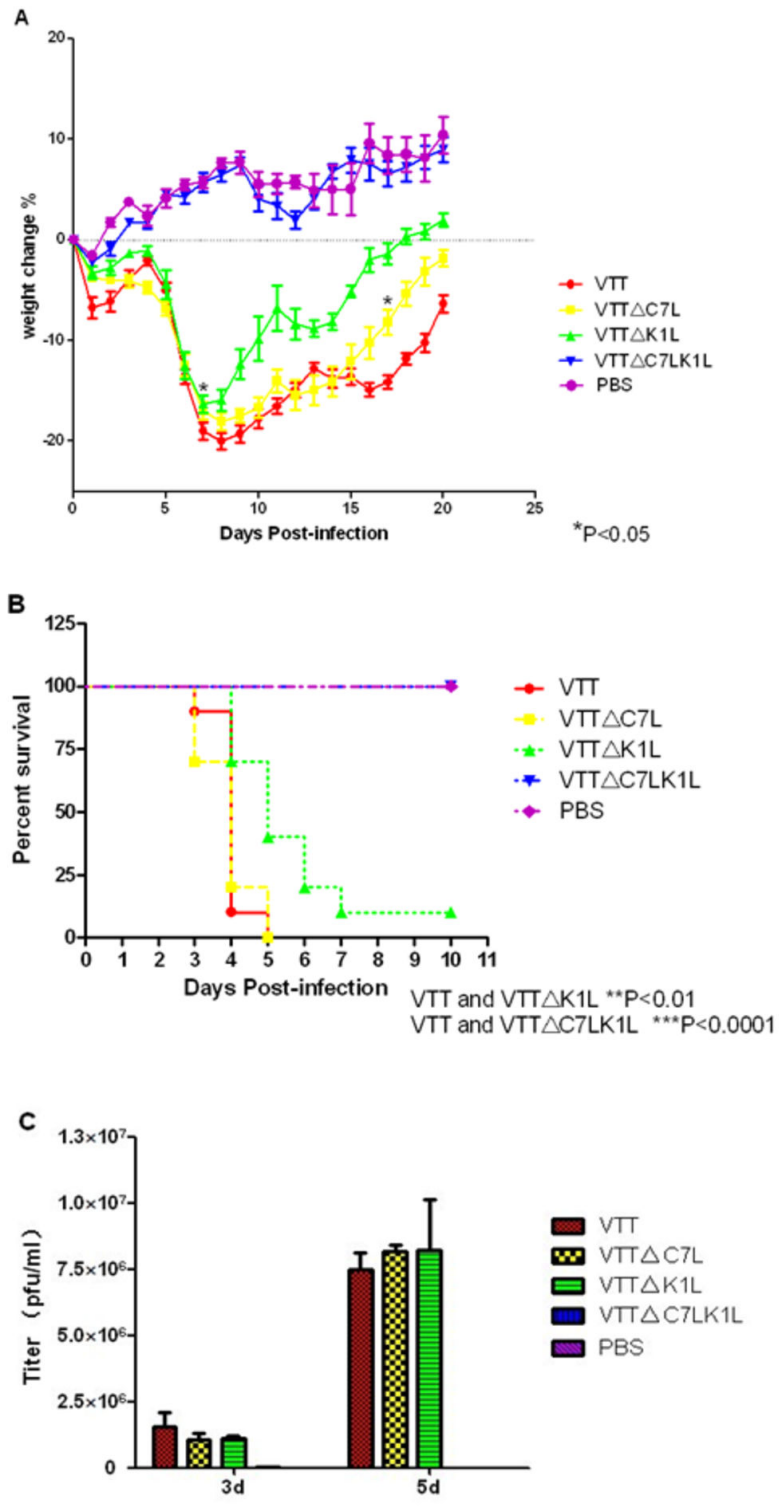
Virulence analysis in BALB/c mouse. (A) Groups of 6 BALB/c mice were infected intranasally with 10^5^ pfu of indicated viruses or mock infected with the same volume of PBS. The mice were then monitored daily for their body weights. For each group, the average and standard deviation of the percent of body weight change are shown. (B) 3 week-old mice were inoculated intracranially with 3×10^4^ pfu viruses, and deaths were recorded daily. The virulence in nervous system decreased when K1L gene was deleted. (C) 3 week-old mice were inoculated intracranially with 3×10^4^ pfu viruses. The brains were ground and supernatant used in plaque assays, which revealed reduced virulence in VTT△C7LK1L.

To determine the virulence of VTT, VTT△C7L, VTT△K1L and VTT△C7LK1L *in vivo*, six week-old mice were infected intranasally with 10^5^ pfu/20 µl viruses and weighed daily until 20 days p.i. (Figure 3A). As in the PBS group, mice infected with VTT△C7LK1L did not experience apparent body weight loss; in fact, body weight consistently rose from day 3 p.i. onwards, resulting in significant difference with VTT (***P<0.0001), and indicating virulence attenuation in VTT△C7LK1L. VTT△C7L and VTT△K1L performed similarly as wildtype VTT in causing 15%-20% body weight loss from day 7 to day 9 p.i.. However, weight loss was significantly attenuated in VTT△K1L from day 7 to day 20 p.i. compared to VTT (*P<0.05), and from day 17 p.i. onwards in VTT△C7L (*P<0.05).

To determine the impact of C7L and K1L genes on neurovirulence *in vivo*, three week-old female BALB/c mice were injected with 3×10^4^ pfu/20 µl VTT, VTT△C7L, VTT△K1L or VTT△C7LK1L intracranially. The survival rate shows that except VTT△C7LK1L and PBS group, mice in VTT, VTT△C7L and VTT△K1L groups began to die from day 3 p.i.: all mice died before day 6 p.i. in VTT and VTT△C7L groups, and only one mouse in VTT△K1L group survived until day 10 p.i. (Figure 3B). Half of the mice died by day 4 p.i. in VTT and VTT△C7L groups, and by day 5 p.i. in VTT△K1L group was. There was no significant difference between VTT and VTT△C7L groups (P>0.05), while neurovirulence decreased greatly in VTT△K1L group (**P<0.01) and VTT△C7LK1L group(***p<0.001).

The brains of mice infected with these vectors were removed on day 3 and day 5 p.i., homogenized, and titrated on CEF cells (Figure 3C). The results showed that VTT△C7L or VTT△K1L had similar titers in the brain to those with VTT on day 3 and 5 p.i., with peak viral replication on day 5 p.i., at titers up to 8×10^6^ pfu/ml. The brain viral titers of mice infected with VTT△C7LK1L decreased from 2.5×10^4^ pfu/ml on day 3 to 1.7×10^3^ pfu/ml on day 5 p.i., which are significantly decreased compared to those in VTT (***P<0.001).

### Deletion of K1L decreases VTT’s cutaneous virulence on rabbit skin

**Figure 4:**
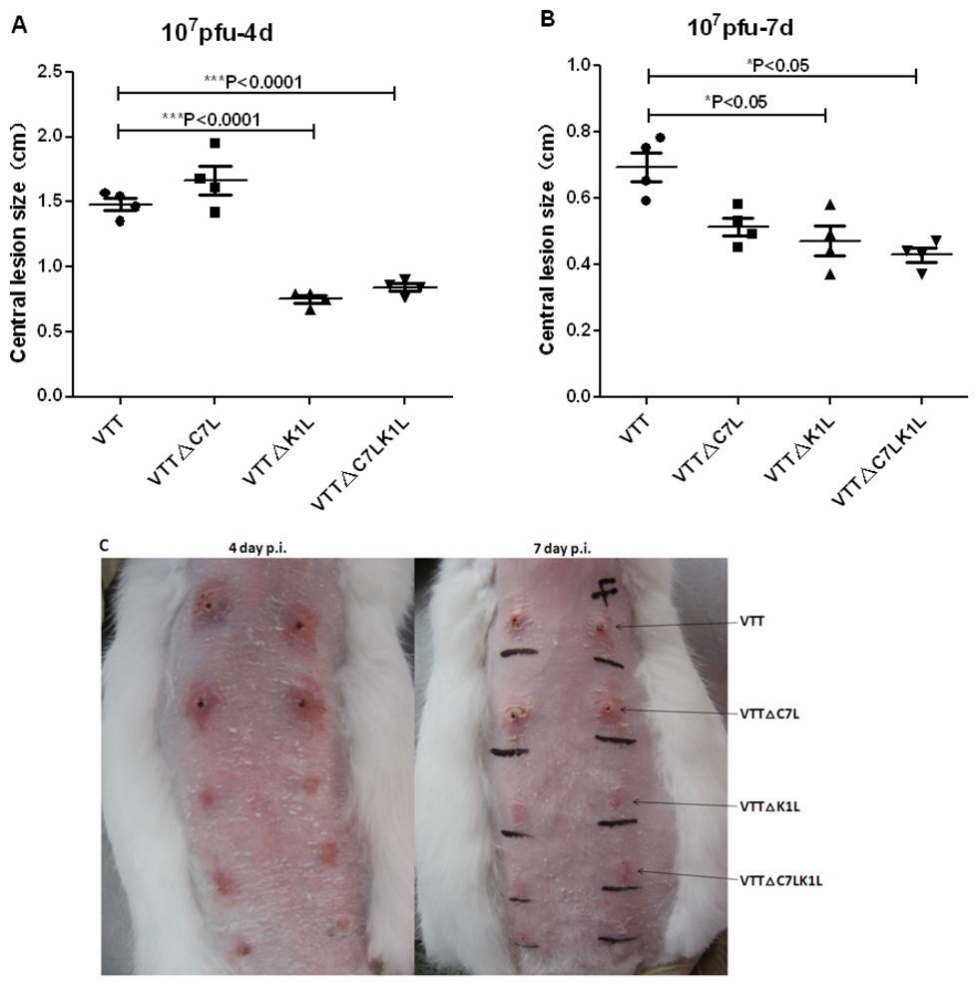
Virulence analysis in rabbit model. Cutaneous virulence in rabbits injected intradermally on the dorsal spine with 10-fold dilution of viruses. Ulcer diameters were recorded at day 4 and day 7 post-infection. (A) The size of lesions formed by 10^7^ pfu viruses were shown at day 4 post-infection. (B) The size of lesions formed by 10^7^ pfu viruses were shown at day 7 post-infection. (C) Deletion of the K1L gene resulted in notable decrease in virulence.

The virulence of VTT, VTT△C7L, VTT△K1L or VTT△C7LK1L on rabbit skin was examined by measuring cutaneous lesions at the inoculation sites on day 4 and day 7 post-infection. Figure 4A and Figure 4B shows the size of erythema caused by 10^7^ pfu VTT and the mutant viruses, and the biggest central lesions appeared on day 4 p.i. Although the lesions formed by VTT△C7L reached 1.67 cm, a little bigger than those formed by VTT (1.48 cm) on day 4 p.i. (Figure 4A), erythemas caused by VTT△C7L were smaller than those caused by VTT on day 7 p.i. (Figure 4B). The size of lesions formed by 10^7^ pfu of VTT△K1L and VTT△C7LK1L were 0.75 cm and 0.84 cm on day 4 p.i., significantly smaller than those caused by parental VTT (***p<0.001); similarly, lesion size were 0.47 cm and 0.42 cm on day 7 p.i., also significantly smaller than VTT formed lesions (*p<0.05). Moreover, 10^4^, 10^5^ and 10^6^ pfu of VTT△K1L and VTT△C7LK1L did not result in observable lesions, while VTT and VTT△C7L did (data not shown).

### Deletion of C7L or K1L decreases vaccinia-specific humoral responses but has no effect on cellular immune responses in BALB/c mice

**Figure 5:**
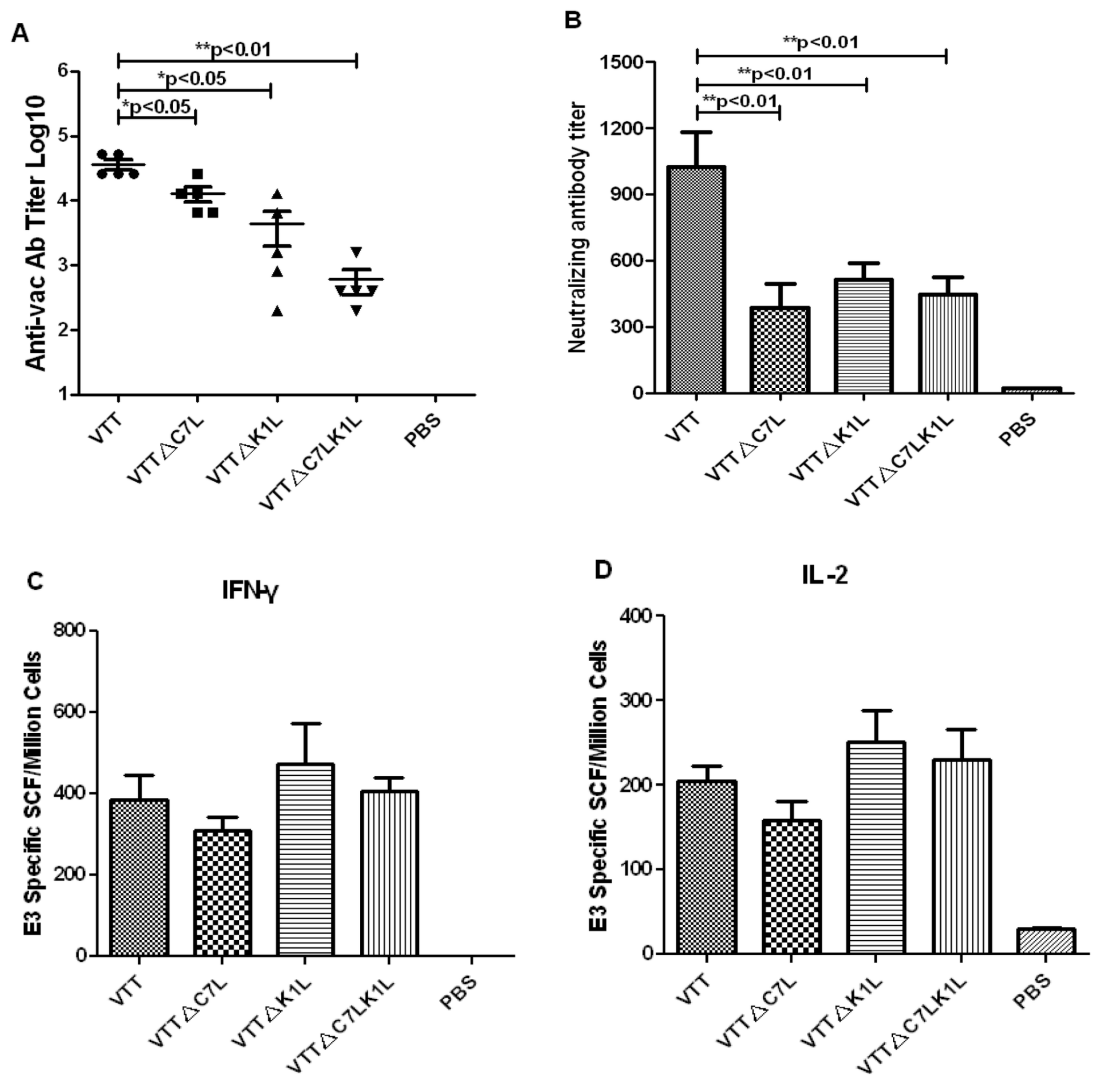
Cellular and humoral responses elicited against VTT in mice. BALB/c mice were immunized with wildtype and deletion mutant VTT, and Mice were sacrificed at week 4 post-infection. (A) Titers of binding antibody elicited by the deletion mutants were notably decreased. (B) Neutralizing antibody titers were defined as the reciprocal of serum dilution that reduced viral plaques by 50%, and the deletion mutants were notably decreased. (C) E3 specific IFN-γ secreting cells immune responses were not significantly different among viruses. (D) E3 specific IL-2 secreting cells immune responses were not significantly different among viruses.

To detect the influence of host range gene deletion on immunogenicity, six week-old mice were injected intramuscularly with 10^7^ pfu of VTT, VTT△C7L, VTT△K1L or VTT△C7LK1L, and vaccinia virus-specific cellular and humoral immune responses were examined. Compared with wildtype VTT, vaccinia-specific binding antibodies induced by VTT△C7L, VTT△K1L or VTT△C7LK1L were decreased by 2.8-fold, 8.2-fold and 59.7-fold, respectively (Figure 5A). The neutralizing antibody titer against a second VTT injection was also evaluated. Consistent with the binding antibodies, neutralizing antibody responses induced by the deletion mutants were also remarkably lower than that induced by VTT by 2 to 3 fold (**P<0.01) (Figure 5B). The magnitude of E3-specific T-cell immune responses induced by VTT△C7L, VTT△K1L or VTT△C7LK1L was not statistically different from that of VTT (P>0.05). In all group, the IFN-γ-secreting T cells reached ~400 Spot forming Cell (SFC) per million cells, and IL-2-secreting T cells reached ~200 SFC per million cells, respectively (Figure 5C, 5D).

C7L and K1L deleted VTT recombinants with HIV genes induced lower vaccinia specific binding antibody responses but had no effect on cellular immune responses in BALB/c mice.

**Figure 6:**
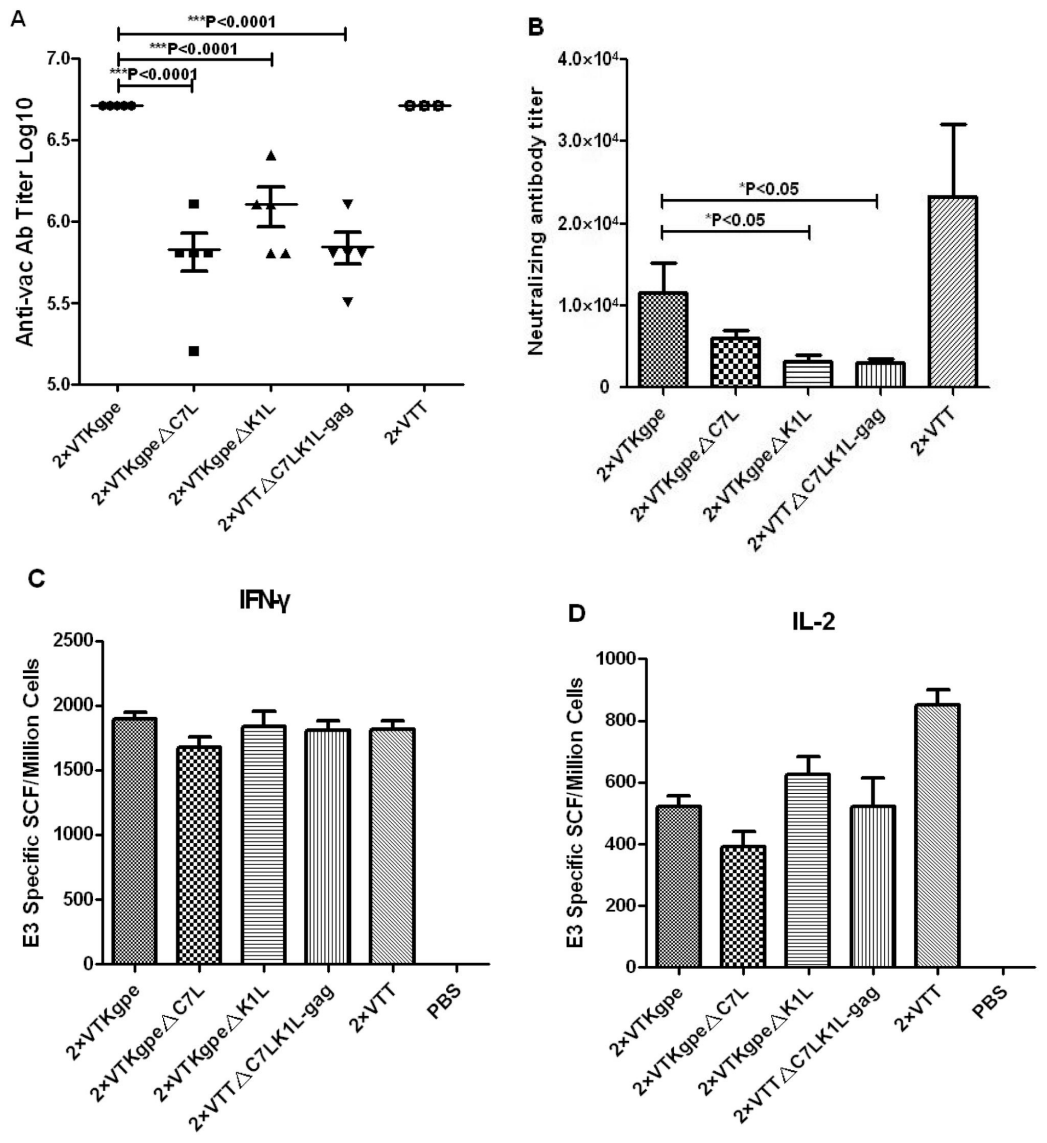
Comparison of cellular response and humoral response after immunized with recombinant mutants and VTKgpe. BALB/c mice were immunized with recombinant mutants and VTKgpe, one week after the final vaccination, mice were sacrificed. (A) Serum was tested for vaccinia specific binding antibody. (B) Serum was tested for vaccinia specific neutralizing antibody. (C) E3 specific IFN-γ secreting cells were not significantly different among viruses. (D) E3 specific IL-2 secreting cells were not significantly different among viruses.

To evaluate the humoral and cellular immunogenicity of HIV gene-carrying VTT mutant vectors, mice were immunized with VTKgpe, VTKgpe△C7L, VTKgpe△K1L or VTT△C7LK1L-gag. The vaccinia specific binding antibody titer of group VTKgpe reached 5×10^6^ (Figure 6A). However, compared with VTKgpe, antibody titers were reduced by 7.6-fold, 4-fold and 7.3-fold in the VTKgpe△C7L, VTKgpe△K1L and VTT△C7LK1L-gag groups, respectively, and the differences were statistically significant (***P<0.0001). Compared with VTKgape, neutralizing antibody responses induced by VTKgpe△K1L or VTT△C7LK1L-gag were also remarkably lower by 3.5-fold or 3.8-fold (Figure 6B). E3-specific T-cell immune responses elicited by VTKgpe△C7L, VTKgpe△K1L or VTT△C7LK1L-gag were comparable to that by VTKgpe (Figure 6C, 6D), with no statistical difference between all groups (P>0.05).

### C7L and K1L deleted VTT recombinants with HIV genes induced strong cellular and humoral immune responses against HIV genes in BALB/c mice

**Figure 7:**
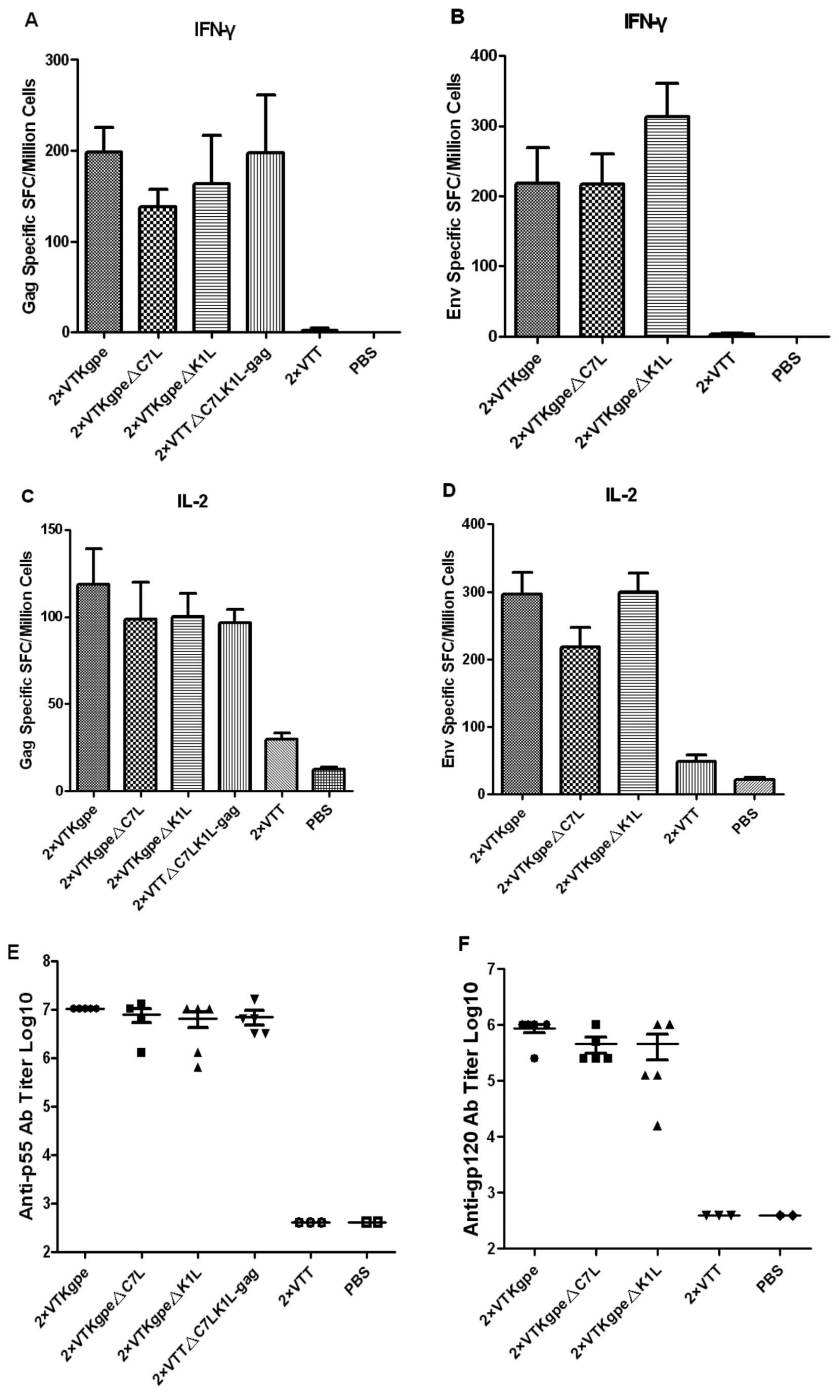
Comparison of cellular response and humoral response against HIV after immunized with recombinant mutants and VTKgpe. Comparison of cellular and humoral responses after mice were immunized with VTKgpe, VTKgpe△C7L, VTKgpe△K1L and VTT△C7LK1L-gag. Mice were sacrificed one week post-infection, and splenocytes were harvested. (A) The gag specific IFN-γ secreting cells were quantified by ELISPOT assay. (B) The env specific IFN-γ secreting cells were quantified. (C) The gag specific IL-2 secreting cells were quantified. (D) The env specific IL-2 secreting cells were quantified. (E) Serum were harvested and tested for p55-specific antibody levels by ELISA assay. (F) Serum was tested for gp120-specific antibody levels.

After assessing the ability of VTT mutants to induce vaccinia-specific responses, we further investigate their immunogenicity for responses against HIV genes. HIV gag and env-specific T-cell responses in mice were determined by IFN-γ and IL-2 ELISPOT assays. The gag-specific IFN-γ responses induced 150-200 SFC per million cells in VTKgpe, VTKgpe△C7L, VTKgpe△K1L and VTT△C7LK1L-gag groups (Figure 7A), and env-specific responses reached 200-300 SFC per million cells in all groups (Figure 7B). No significant differences were observed in magnitude between the VTKgpe△C7L, VTKgpe△K1L or VTT△C7LK1L-gag groups and the VTKgpe group. The gag-specific IL-2 responses induced 100-120 SFC per million cells in all groups (Figure 7C); env-specific responses reached 220 SFC per million cells in VTKgpe△C7L, a little lower than those in VTKgpe and VTKgpe△K1L groups (Figure 7D), but the differences were not statistically significant.

Gag and Env specific antibody responses of the mice immunized with VTKgpe△C7L, VTKgpe△K1L or VTT△C7LK1L-gag were also evaluated. The humoral responses to HIV-1 gag (p55) antigen induced by VTKgpe is 1×10^7^ average serum reciprocal titer, slightly higher than those elicited by VTKgpe△C7L, VTKgpe△K1L or VTT△C7LK1L-gag (Figure 7E). VTKgpe induced the average serum reciprocal titer of 1×10^6^ against env (gp120) antigen, twice as high as that induced by VTKgpe△C7L or VTKgpe△K1L. However, the differences among all groups were not statistically significant (Figure 7F).

## Discussion

The replicating vaccinia Tian Tan strain (VTT) has been used extensively in China as a smallpox vaccine, and the HIV vaccine based on this vector has been initiated for Phase II clinical trials. However, the strain is known to cause side effects in mice and rabbit, such as body weight loss and skin damage [13,14,29]. Currently, several highly attenuated vaccinia strains have been developed as live viral vectors [30–33]. The further attenuation of VTT is therefore desirable for its usage as a vaccine vector in humans.

C7L and K1L genes are host range genes of vaccinia virus, which permit the virus to replicate in various cell substrates *in vitro* [34]. The deletion of K1L and C7L from VACV results in abortive replication in most mammalian cell lines [21,24]. Studies have shown that VTT has broad cell tropism, with the ability to enter 13 cell types [14], despite fact that the the virus was passaged in calf skin or CEFs for many generations during the production of smallpox vaccine [35]. In this study, we constructed a series of K1L and C7L deleted VTT mutants: VTT△C7L, VTT△K1L and VTT△C7LK1L. As previous study shows, a mutant VTT strain with deletion of the M1L to K2L gene region (which includes K1L) replicated well in BHK-21 and Vero cell lines, but was abortive in HeLa cells [15]. When the C7L to K2L gene region (which includes C7L and K1L) were deleted, the mutant VTT did not produce cytopathic effect in Vero cells, but was capable of growing in the BHK-21 cells [29]. Our studies show that, in the absence of the K1L gene, the replicative ability of VTT was restricted in HeLa cells, while C7L gene was required for VTT to replicate and disseminate in BHK-21 cells. Similar to the observation that genes in the C7L-K2L region restrict VTT replication in Vero cells, deletion of C7L and K1L in VTT leads to aborted viral replication in Vero cells and reduced infectivity in human and murine cells (Figure 2). The growth kinetics of VTKgpe, VTKgpe△C7L, VTKgpe△K1L and VTT△C7LK1L-gag were also monitored in CEF, BHK-21, Vero and HeLa cell lines, they behave as same as VTTs (Figure S2).

Safety in humans is a priority issue for the VTT-based HIV vaccine candidate, and its safety in immunodeficient individuals is particularly important. The VTT mutant with C7L-K2L genes deleted generated mild signs of illness in intranasally and intracranially infected mice, as well as in rabbits [29]. In our study, BALB/c mice infected with VTT△C7LK1L did not lead to apparent body weight loss, while intranasal infection by wildtype VTT caused about 20% loss (Figure 3A). Meanwhile, intracranial infection with VTT△C7LK1L resulted in significantly reduced neurovirulence in mice. At the same time, the titer of VTT△K1L was as high as parental VTT on day 3 and day 5 p.i., while the survival curve showed that the neurovirulence of VTT△K1L decreased (Figure 3B, 3C). We infer that the replicate capability of the virus in mouse brain may not be the only reason which can cause death; there may be other systemic effects involved outside of neurovirulence in the brain. However, further studies are needed to identify the exact cause and mechanisms involved. At the same time, considering cutaneous lesions are one of the main side effects of vaccinia virus, we tested the virulence of VTT mutants on the rabbit skin. Cutaneous lesions caused by VTT△K1L and VTT△C7LK1L were not observed until the virus titer was increased to 10^7^ pfu, and even then did not result in ulceration (Figure 4). It is reported that K1L is a host range gene that is essential for vaccinia viral replication in RK13 (rabbit kidney) cells [21,36,37]. Our *in vivo* results confirm that K1L is an essential gene for VTT in rabbit skin cells.

In sum, VTT△K1L results in greater attenuation in body weight loss and skin virulence than does VTT△C7L. These results are consistent with former studies, which show that these two host range genes play different roles in identifying host cells of different genus and facilitating their infection [38–40]. The poxvirus vectors NYVAC and MVA, which have C7L and/or K1L deletions, were highly attenuated and served as a safety standard for poxvirus vectors in human vaccine development [41,42]. VTT△C7LK1L is the most attenuated vector among our three mutants, with reduced virulence in both the nervous system and epidermal tissue, and may be a promising candidate for safe VTT use in human vaccines.

Aside from virulence attenuation, a good vaccine candidate must elicit strong immunogenicity against foreign antigens. Our HIV gene-carrying host range gene-deleted recombinants VTKgpe△C7L, VTKgpe△K1L and VTT△C7LK1L-gag elicited comparably strong cellular and humoral immune responses as did the wildtype-derived VTKgpe (Figure 7). This indicates that C7L and K1L deletion did not affect the immune responses against the target foreign antigens, namely HIV genes. Moreover, the vaccinia specific binding antibody response decreased notably when C7L and K1L were deleted, especially after a vaccinia boost (Figures 5A, 6A). Significant differences were also observed in vaccinia neutralizing antibody (Figures 5B, 6B). The reduction of humoral responses against the vector itself may make possible a multi-vaccinia boost strategy in the vaccination against HIV.

Since most of the poxvirus vector is used together with other vaccine components in a prime boost manner, we evaluated HIV-specific cellular and humoral responses from mice primed with DNA vaccine at week 0, and boosted with VTKgpe, VTKgpe△C7L, VTKgpe△K1L or VTT△C7LK1L-gag at week 4, however the results were not as high as we expected (Figure S3). The further experiments are under way for more effective immunology strategies.

VTT as the vector for HIV vaccine has gone stepped into phase II clinical trials in China. Along with the widespread application of VTT vaccine, the safer and more effective vaccine vectors are needed in the future. C7L and K1L genes deleted mutants show highly attenuation and reduced immune response against the vector itself in the animal models. It is seemed these host range genes muted vaccinia could contribute to HIV or other virus vaccine in future.

## Supporting Information

Figure S1 Sequences of adjacent genes harboring the deletions. (A) Sequences of C6L gene in four strains harboring C7L gene. (B) Sequences of C8L gene in four strains harboring C7L gene. (C) Sequences of K2L gene in four strains harboring K1L gene. (D) Sequences of M2L gene in four strains harboring K1L gene.
(TIF)

Figure S2 Growth curves of the recombinant VTKgpes in vitro. (A) Growth curves of the recombinant VTKgpes in CEF cells at MOI of 0.05 pfu/cell. Virus yields at 0, 6, 12, 24, 36 and 48 h pi were determined by plaque assay on the permissive CEF cells. (B) Growth curves of the recombinant VTKgpes in BHK-21 cells at MOI of 0.05 pfu/cell. Virus yields were determined as the former. (C) Growth curves of the recombinant VTKgpes in Vero cells at MOI of 0.05 pfu/cell. Virus yields were determined as the former. (D) Growth curves of the recombinant VTKgpes in HeLa cells at MOI of 0.05 pfu/cell. Virus yields were determined as the former.
(TIF)

Figure S3 Comparison of cellular response and humoral response after primed with DNA and boosted with recombinant mutants. BALB/c mice were immunized with DNA and recombinant mutants, one week after the final vaccination, mice were sacrificed. (A) Serum was tested for p55 specific binding antibody. (B) The gag specific IFN-γ secreting cells were quantified by ELISPOT assay.
(TIF)
