# Supplementary figures and images for: Deletion of C7L and K1L Genes Leads to Significantly Decreased Virulence of Recombinant Vaccinia Virus TianTan
Source: PLoS One. 2013 Jul 1;8(7):e68115. doi: 10.1371/journal.pone.0068115 (PMC3698190; doi:10.1371/journal.pone.0068115)

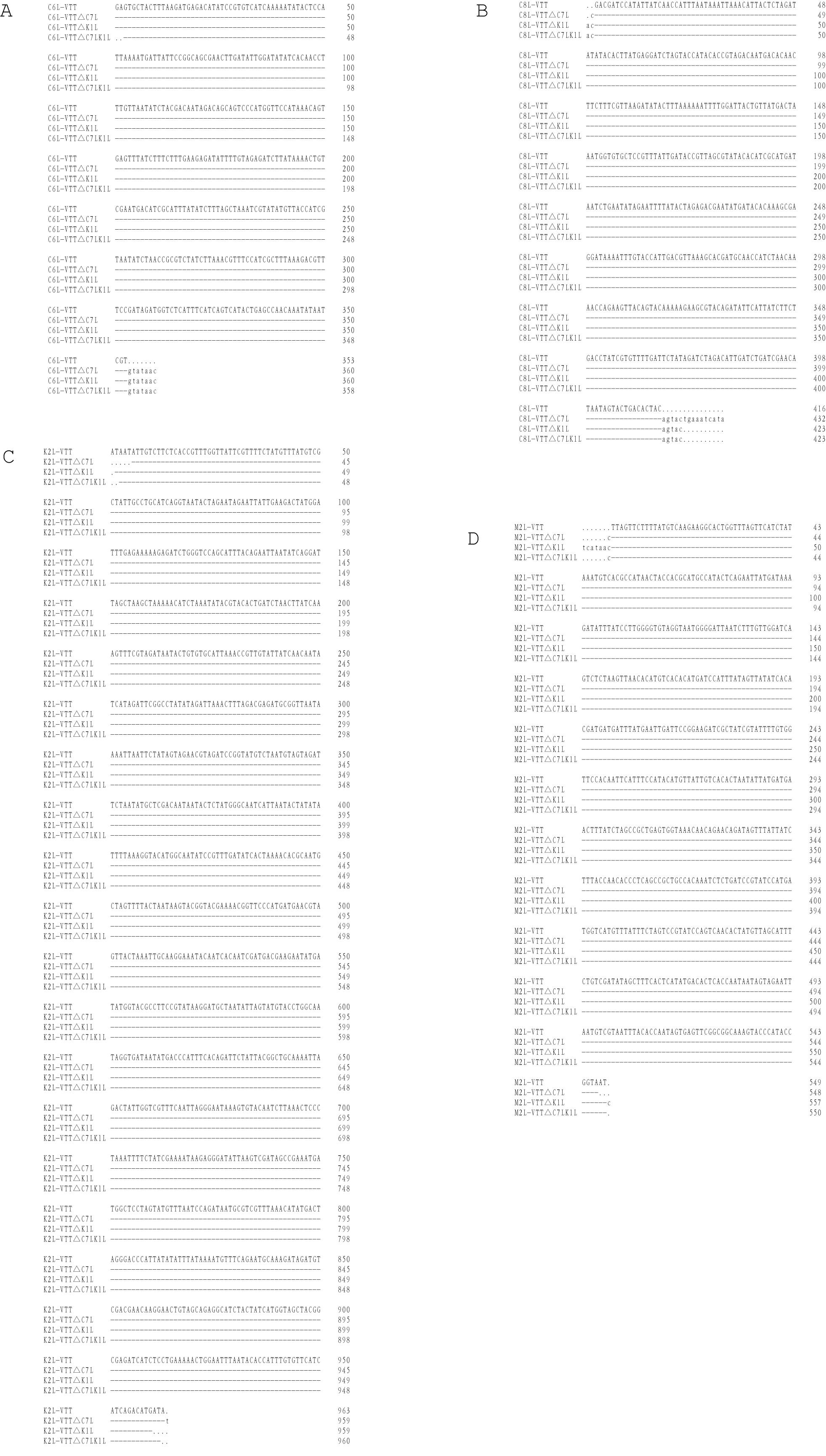

Supplement: Figure S1 — (A) Sequences of C6L gene in four strains harboring C7L gene. (B) Sequences of C8L gene in four strains harboring C7L gene. (C) Sequences of K2L gene in four strains harboring K1L gene. (D) Sequences of M2L gene in four strains harboring K1L gene. (TIF) [file pone.0068115.s001.tif]

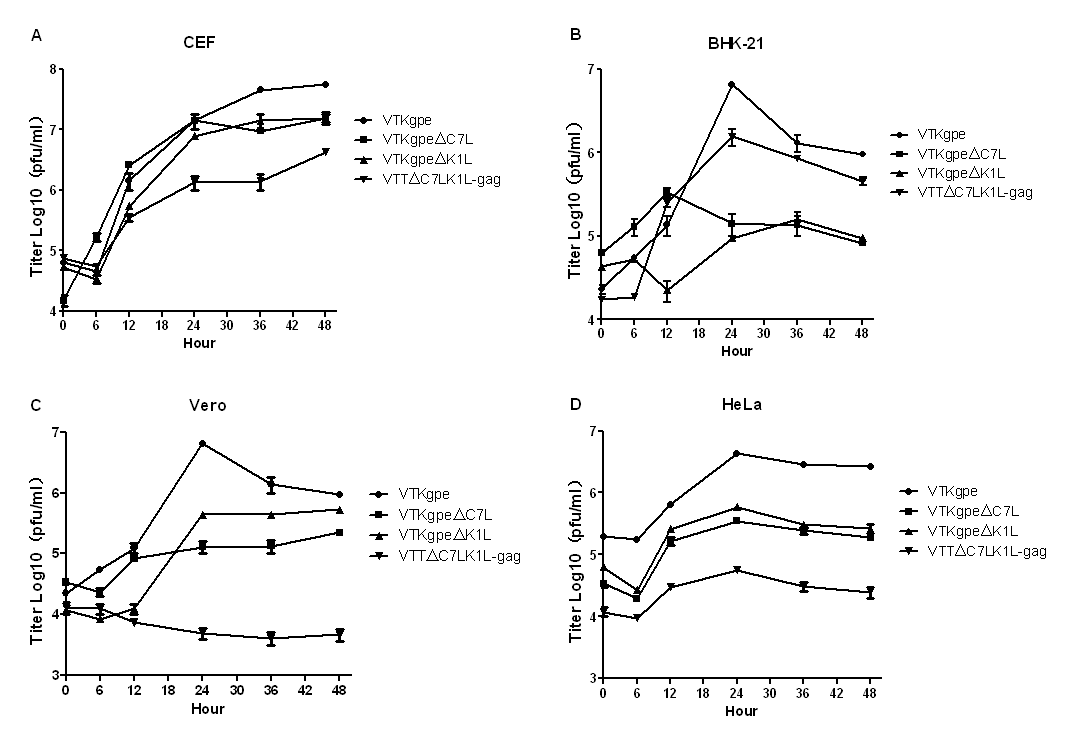

Supplement: Figure S2 — (A) Growth curves of the recombinant VTKgpes in CEF cells at MOI of 0.05 pfu/cell. Virus yields at 0, 6, 12, 24, 36 and 48 h pi were determined by plaque assay on the permissive CEF cells. (B) Growth curves of the recombinant VTKgpes in BHK-21 cells at MOI of 0.05 pfu/cell. Virus yields were determined as the former. (C) Growth curves of the recombinant VTKgpes in Vero cells at MOI of 0.05 pfu/cell. Virus yields were determined as the former. (D) Growth curves of the recombinant VTKgpes in HeLa cells at MOI of 0.05 pfu/cell. Virus yields were determined as the former. (TIF) [file pone.0068115.s002.tif]

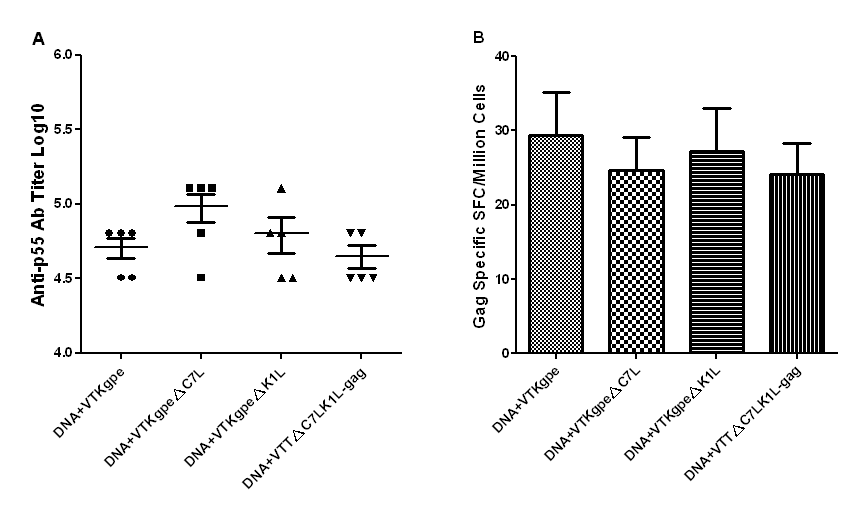

Supplement: Figure S3 — BALB/c mice were immunized with DNA and recombinant mutants, one week after the final vaccination, mice were sacrificed. (A) Serum was tested for p55 specific binding antibody. (B) The gag specific IFN-γ secreting cells were quantified by ELISPOT assay. (TIF) [file pone.0068115.s003.tif]
